# Total knee arthroplasty: good agreement of clinical severity scores between patients and consultants

**DOI:** 10.1186/1471-2474-7-61

**Published:** 2006-07-31

**Authors:** Ananthan D Ebinesan, Bhupinder S Sarai, Gayle Walley, Stephen Bridgman, Nicola Maffulli

**Affiliations:** 1 University of Keele, Department of Postgraduate Medicine, Thornburrow Drive, Hartshill, Stoke-on-Trent, Staffordshire, UK; 2 Department of Trauma and Orthopaedic Surgery, Keele University School of Medicine, University Hospital of North Staffordshire, Thornburrow Drive, Hartshill, Stoke on Trent, Staffordshire, UK; 3 Newcastle-under-Lyme Primary Care NHS Trust, Directorate of Public Health, Civic offices, Merrial Street, Newcastle-under-Lyme, UK

## Abstract

**Background:**

Nearly 20,000 patients per year in the UK receive total knee arthroplasty (TKA). One of the problems faced by the health services of many developed countries is the length of time patients spend waiting for elective treatment. We therefore report the results of a study in which the Salisbury Priority Scoring System (SPSS) was used by both the surgeon and their patients to ascertain whether there were differences between the surgeon generated and patient generated Salisbury Priority Scores.

**Methods:**

The Salisbury Priority Scoring System (SPSS) was used to assign relative priority to patients with knee osteoarthritis as part of a randomised controlled trial comparing the standard medial parapatellar approach versus the sub-vastus approach in TKA. The operating surgeons and each patient completed the SPSS at the same pre-assessment clinic. The SPSS assesses four criteria, namely progression of disease, pain or distress, disability or dependence on others, and loss of usual occupation. Crosstabs and agreement measures (Cohen's kappa) were performed.

**Results:**

Overall, the four SPSS criteria showed a kappa value of 0.526, 0.796, 0.813, and 0.820, respectively, showing moderate to very good agreement between the patient and the operating consultant. Male patients showed better agreement than female patients.

**Conclusion:**

The Salisbury Priority Scoring System is a good means of assessing patients' needs in relation to elective surgery, with high agreement between the patient and the operating surgeon.

## Background

Nearly 20,000 patients per year in the UK receive total knee arthroplasty (TKA) at an estimated annual cost of 70 million pounds. Patients who undergo knee surgery are affected by their condition on a very personal basis in terms of disability. For athletes at the prime of their career, not being able to perform at an optimal level competitively is a major disability. In comparison, an elderly patient with knee osteoarthritis may find simple activities such as walking to be a disability [1].

One of the problems faced by the health services of many developed countries is the length of time patients spend waiting for elective treatment. Despite various efforts, waiting lists on the United Kingdom National Health Service (NHS) remain long, with the various clinical specialities having different waiting times for elective treatment [2]. The focus of the government on the length of time spent waiting and on the total numbers of patients on NHS waiting lists may have sidelined the need to treat patients according to their clinical need [3]. In the UK, non-urgent or elective health care is accessed via General Practitioner referrals for an outpatient consultation. Thereafter, a decision is made to offer the patient a booked admission date or is placed on an inpatient waiting list [2].

**Figure 1:**
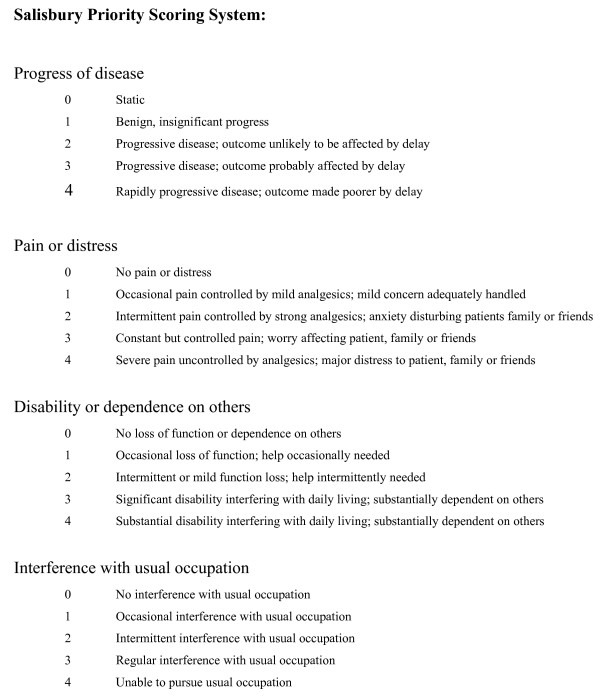
Salisbury scoring system point scores and descriptions.

Rating systems should provide an objective assessment of any given subject so that direct comparison can be made with other examples of the same condition [4]. A total of 34 different rating systems to assess patients for TKA have been used between 1972 and 1992 [5]. At present, it is not clear how surgeons and patients requiring total knee arthroplasty compare in relation to the perceived need for surgery. We therefore report the results of a study in which the Salisbury Priority Scoring System (SPSS) (see figure 1) was used by both the surgeon and their patients to ascertain whether there were differences between the surgeon generated and patient generated Salisbury Priority Scores. Through this study we were also able to identify if the SPSS was a good tool to prioritise patients requiring TKA.

## Methods

A randomised controlled trial, the SMAK Arthroplasty trial, compared the effectiveness and efficiency of a standard medial parapatellar approach versus a sub-vastus approach in TKA and evaluated clinical outcomes of care in each group. Patients undergoing primary bi-(tibial-femoral joint) or tri-(tibial-femoral-patella) compartmental knee replacement for any indications were recruited from the University Hospital of North Staffordshire. Ethical approval was obtained from the local ethics committee for the trial.

Patients were recruited at their pre-operative assessment, with randomisation taking place on the day of admission for surgery. Only patients who required unilateral knee replacement who had given their informed consent to take part in the study were eligible to take part in the trial. The operating surgeon also had to have no clear preference for either of the approaches. Patients not eligible to enter the trial were those who needed revision knee replacement, bilateral knee replacement, required major arthrotomy in the other knee within a 12 month period, had previous open surgery in or around the knee over the last 12 months, or had a valgus angle greater than 20°.

As part of this trial, the operating surgeon and the patient completed the Salisbury Priority Scoring System (SPSS) (see figure 1). This scoring system enables consultants to assign relative priority to patients for elective healthcare [6]. At the pre-assessment clinic, patients were assigned points according to clinical and social criteria to reflect their 'need' for treatment. The SPSS examines aspects of the patient's condition requiring total knee arthroplasty, and a score of 0 to 4 is given for each criterion. Points are assigned to reflect the rate of progress of their disease, pain or distress, disability or dependence on others, and loss of usual occupation.

All 230 patients recruited for the trial and the clinicians performing the procedure completed the SPSS at the pre-assessment clinic. The research nurse involved in the SMAK arthroplasty trial facilitated both groups in filling out the questionnaire separately. Analyses of quantitative results were undertaken using the Statistical Package for Social Sciences (SPSS Manager 12.0 for Windows). Crosstabs was performed to compare the agreement between clinicians and patients. Cohen's kappa (*K*) values were obtained to measure the agreement between the two sets of data.

## Results

There was moderate to very good agreement in the overall data between patients and clinicians in the study. For the first criterion, progress of disease, a *K *value of 0.526 was obtained. This indicates a moderate agreement between clinicians and patients. The second criterion, pain or distress, produced a *K *value of 0.796, which shows good agreement between clinicians and patients. The criterion disability of dependence on others had a *K *value of 0.813, indicating very good agreement. Finally, the fourth criterion, interference with usual occupation, had a *K *value of 0.820, again indicating very good agreement between clinicians and patients.

When the data were analysed based on patients' gender, apart from the first criterion, male patients had a higher agreement level compared to female patients. For male patients, the first criterion had a *K *value of 0.472, a moderate level of agreement. The *K *values for the second, third, and fourth criterion were 0.824, 0.844, and 0.858 respectively. These values showed a very good agreement between the patients and clinicians. In comparison, females produced moderate to good levels of agreement with *K *values of 0.589, 0.768, 0.781, and 0.774 for the individual criterion. Overall, clinicians and male patients show a better level of agreement.

## Discussion

Clinicians and patients show good agreement in regards to their need for TKA, with two of the four criteria of the SPSS showing very good agreement. Good communication between the two groups is essential to meet the needs of patients that present for elective orthopaedic healthcare. Steps should be taken to enable patients to be treated without having to wait on an arbitrary waiting list. Patients should be treated on a needs basis, rather than be put on a queue and told to wait in line.

The simplicity of the SPSS enables consultants and patients to reach a good agreement. The four-item questionnaire imposed very little burden on the patients and the consultants, making it easy for them to complete it. This is not the case with other widely used health status questionnaires [7]. With easy to understand questions and objective assessments of the condition the patient is suffering from, both patients and clinicians are able to communicate sufficiently to reach a good consensus in regards to the condition being treated. The level of agreement seen between patients and clinicians shows that clinicians are able to understand sufficiently well how patients feel in regards to their knee osteoarthritis. Good communication will enable better decision-making, and a higher level of patient satisfaction.

With the introduction of priority-scoring systems, the management of waiting lists becomes more transparent, with priority given based on explicit criteria, with patients being treated on a needs basis and not just on time already waited [6].

One of the most serious issues that might emerge and limit the potential benefit of such a system is the possibility of fraud. This has implications on every level of the care pathway. Clinicians will have a greater say in prioritising the patients, and such a system will allow for favouritism to take place. Consultants will have more say in regards to their operating lists, as the final decision will lie with them. Priority scoring systems will also fail to discern between high and low priority cases if patients wise up to the system and learn to work their way around it. Clinicians will also be more pushed into making a decision if they were more sympathetic towards patients' need. Difficult patients who harass clinicians by wanting surgery could also have an implication on the system, by demanding surgery.

As part of this study we analysed gender differences between male and female patients. Male patients and clinicians showed a better level of agreement. One possible explanation for this is the suggestion that women have a higher depression rate than men [8], and there is a link between depression and symptom perception [9]. This would lower the threshold for complaint in regards to their requirement for a TKA, making it slightly more difficult for clinicians to assess the scenario objectively. The high proportion of male consultants in orthopaedics could also be another contributing factor in failure to communicate adequately. Currently, outcome measures such as the Oxford Knee Score are used in clinical practise. Prioritising patients using the Oxford Knee Score is possible, but the SPSS has an advantage from a practicality point of view. The SPSS is a much simpler and easy to use tool. It requires very little time and effort to complete, making it ideal for clinicians to use in a busy outpatient clinic environment. Prioritising patients can be done from the very first encounter by clinicians and this can be reassessed again by completing the same system when reviewing patients. Furthermore, the Oxford Knee score looks at outcome measures as well and isn't purely a prioritisation tool. The SPSS was not designed specifically for TKA, but due to its simplicity and easily understood criteria, it was utilised without too much difficulty and produced good agreement between patients and clinicians.

One limitation of the study was that the SPSS was not compared directly with another scoring system such as the Oxford Knee Score during the consultation. As such, no head to head comparison between the SPSS and another priority scoring system was made. Doing it retrospectively would not produce an accurate representation of the patient and clinician's perception with regards to their condition requiring surgery**. **It will however be of value in the future to conduct another study to determine how discriminatory the SPSS is in comparison with another severity scoring system by ensuring clinicians and patients fill out both scoring systems at the same time.

## Conclusion

In conclusion, there is high agreement between patients' scores and surgeons' scoring of patients with the Salisbury Priority Scoring System. The implementation of a priority scoring system should be considered in elective health care settings. A more definitive and specific questionnaire, used only for TKA surgery needs to be developed to aid in prioritising patients, rather than placing them on arbitrary waiting lists. A similar but specific system can then be further implemented in other areas with long waiting lists. The key to ensuring that such a system is easily implemented and is successful is to keep it simple and accurate. The balance between discretion by clinicians or flexibility and avoidance of fraud by any party needs to be maintained for this system to work efficiently and successfully.

## Competing interests

The author(s) declare that they have no competing interests.

## Authors' contributions

All authors contributed equally to this work. NM is the corresponding author. All authors read and approved the final manuscript.

## Pre-publication history

The pre-publication history for this paper can be accessed here:
